# Associations between Radiomics and Genomics in Non-Small Cell Lung Cancer Utilizing Computed Tomography and Next-Generation Sequencing: An Exploratory Study

**DOI:** 10.3390/genes15060803

**Published:** 2024-06-18

**Authors:** Alessandro Ottaiano, Francesca Grassi, Roberto Sirica, Emanuela Genito, Giovanni Ciani, Vittorio Patanè, Riccardo Monti, Maria Paola Belfiore, Fabrizio Urraro, Mariachiara Santorsola, Alfonso Maria Ponsiglione, Marco Montella, Salvatore Cappabianca, Alfonso Reginelli, Mario Sansone, Giovanni Savarese, Roberta Grassi

**Affiliations:** 1 Istituto Nazionale Tumori di Napoli, IRCCS “G. Pascale”, 80131 Naples, Italy; a.ottaiano@istitutotumori.na.it (A.O.); mariachiara.santorsola@istitutotumori.na.it (M.S.); 2 Department of Precision Medicine, Università degli Studi della Campania “Luigi Vanvitelli”, 80138 Naples, Italy; emanuela20112003@hotmail.it (E.G.); dott.giovanniciani@gmail.com (G.C.); patavittorio@gmail.com (V.P.); riccardo.monti@unicampania.it (R.M.); mariapaola.belfiore@unicampania.it (M.P.B.); fabrizio.urraro@uicampania.it (F.U.); salvatore.cappabianca@unicampania.it (S.C.); alfonso.reginelli@unicampania.it (A.R.); roberta.grassi@unicampania.it (R.G.); 3 AMES—Centro Polidiagnostico Strumentale, SRL, 80013 Naples, Italy; roberto.sirica@centroames.it (R.S.); giovanni.savarese@centroames.it (G.S.); 4 Department of Electrical Engineering and Information Technology, University of Naples “Federico II”, 80125 Naples, Italy; alfonsomaria.ponsiglione@unina.it (A.M.P.); mario.sansone.unina@gmail.com (M.S.); 5 Pathology Unit, Department of Mental and Physical Health and Preventive Medicine, Università degli Studi della Campania “Luigi Vanvitelli”, 80138 Naples, Italy; marco.montella@unicampania.it

**Keywords:** radiomics, genomics, non-small cell lung cancer, prognosis, ROS1, ALK

## Abstract

Background: Radiomics, an evolving paradigm in medical imaging, involves the quantitative analysis of tumor features and demonstrates promise in predicting treatment responses and outcomes. This study aims to investigate the predictive capacity of radiomics for genetic alterations in non-small cell lung cancer (NSCLC). Methods: This exploratory, observational study integrated radiomic perspectives using computed tomography (CT) and genomic perspectives through next-generation sequencing (NGS) applied to liquid biopsies. Associations between radiomic features and genetic mutations were established using the Area Under the Receiver Operating Characteristic curve (AUC-ROC). Machine learning techniques, including Support Vector Machine (SVM) classification, aim to predict genetic mutations based on radiomic features. The prognostic impact of selected gene variants was assessed using Kaplan–Meier curves and Log-rank tests. Results: Sixty-six patients underwent screening, with fifty-seven being comprehensively characterized radiomically and genomically. Predominantly males (68.4%), adenocarcinoma was the prevalent histological type (73.7%). Disease staging is distributed across I/II (38.6%), III (31.6%), and IV (29.8%). Significant correlations were identified with mutations of *ROS1* p.Thr145Pro (shape_Sphericity), *ROS1* p.Arg167Gln (glszm_ZoneEntropy, firstorder_TotalEnergy), *ROS1* p.Asp2213Asn (glszm_GrayLevelVariance, firstorder_RootMeanSquared), and *ALK* p.Asp1529Glu (glcm_Imc1). Patients with the *ROS1* p.Thr145Pro variant demonstrated markedly shorter median survival compared to the wild-type group (9.7 months vs. not reached, *p* = 0.0143; HR: 5.35; 95% CI: 1.39–20.48). Conclusions: The exploration of the intersection between radiomics and cancer genetics in NSCLC is not only feasible but also holds the potential to improve genetic predictions and enhance prognostic accuracy.

## 1. Introduction

Non-small cell lung cancer (NSCLC) comprises approximately 80% of all lung cancer cases, with a predominant diagnosis at an advanced stage [1]. Survival outcomes in NSCLC exhibit significant variability depending on the stage at diagnosis. Localized or early-stage disease (Stages I and II) often shows a 5-year survival rate exceeding 50%. Conversely, as the disease progresses to regional lymph nodes (Stage III), the 5-year survival rate diminishes to around 30%. This variability underscores the impact of multidisciplinary treatments encompassing neoadjuvant and adjuvant therapies. In cases of distant or metastatic NSCLC (Stage IV), despite advancements in targeted therapy addressing specific gene alterations and immunotherapy, both in initial and subsequent treatments, the prognosis remains daunting, with 5-year survival rates plummeting below 5% [2]. The growing significance of genetic assessment in NSCLC is driven by both biological and therapeutic considerations [3]. Understanding the biology of this highly heterogeneous tumor group advances in tandem with therapeutic innovation. Consequently, the therapeutic landscape for NSCLC has undergone substantial evolution, notably with the advent of precision therapies tailored for specific gene alterations (e.g., EGFR, BRAF) and rearrangements (e.g., ROS1, ALK) [4]. The targeted approach to these alterations has demonstrated remarkable efficacy, representing a pivotal advancement in NSCLC management along with immune therapies [5]. Furthermore, the exploration of genetic biomarkers is continually advancing, encompassing not only therapeutic considerations but also diagnostic and technological implications.

On the other side, radiomics stands as a burgeoning quantitative analytical paradigm within the realm of medical imaging [6]. This methodology involves extracting and analyzing a plethora of image features within tumors, encompassing both their spatial and temporal heterogeneity. The information gleaned from these features can be utilized to construct models for diagnosis, prognosis, and prediction. Radiomics analysis is applicable across diverse multimodality medical imaging platforms, including ultrasound (US), computed tomography (CT), magnetic resonance (MR), and positron emission tomography (PET) scans [7,8,9,10]. Recent strides in CT analysis technology have enabled a sophisticated quantitative assessment of features and pixel-based textures, particularly in tumor characterization. The integration of machine-learning algorithms into the domain of artificial intelligence (AI) has markedly augmented the capabilities of radiomics [11]. Notably, the application of AI-driven algorithms using CT images has demonstrated substantial efficacy in predicting treatment responses and prognoses [12]. These collaborative advancements underscore the potential of radiomics as a robust tool for the comprehensive analysis of medical imaging data, enhancing clinical decision-making. Among the recent, captivating, and groundbreaking applications of radiomics is its potential to predict or characterize cancer genetics, offering possibilities for biomarker discovery and revealing comprehensive tumor features, including genetic aspects [13].

The synergistic integration of ‘omics’ disciplines, particularly genomics and radiomics, emerges as pivotal for attaining diagnostic and prognostic results that transcend the intrinsic limitations of singular disciplines, enabling a more comprehensive and profound understanding of specific genetic characteristics. In this exploratory investigation, we concurrently characterized a cohort of NSCLC patients using both radiomic perspectives via CT and genomic perspectives through NGS. Utilizing a machine-learning methodology integrated with artificial intelligence, the primary aim was to establish a correlation between CT-derived radiomic features and NGS findings. This approach aimed to unveil an innovative, non-invasive radiomics prediction model for cancer genetics.

## 2. Patients and Methods

### 2.1. Study Design and Patients’ Selection Criteria

This observational exploratory study included all sequentially diagnosed and radiologically staged lung cancer patients treated at an academic center (“Federico II” University) who provided consent for liquid biopsy for genetic assessment before any therapeutic intervention. The study spanned from December 2020 to April 2022, and the pre-therapy genetic assessments were carried out at a single center (Centro Ames). The study was designed to closely mirror real-world patients. Consequently, no selection criteria based on age, comorbidities, or Eastern Cooperative Oncology Group (ECOG) Performance Status were imposed, even though only a minor subset exhibited an ECOG PS of 2. The exclusion criteria encompassed the following several aspects: CT images of subpar quality, characterized by artifacts arising from metallic objects or motion disturbances such as breathing; tumor contours that were indiscernible and unsuitable for CT segmentation due to proximity to obstructive pneumonia and atelectasis; individuals with a history of prior treatment; and those with a life expectancy of less than three months. All patients underwent treatment, including surgery if feasible, or radiotherapy, chemotherapy, or combinations thereof, following the guidelines of the European Society of Medical Oncology (ESMO) [14,15,16].

Prior to treatment administration and molecular pathology assessments, all patients provided written informed consent. The study was approved by the Ethics Committee “Comitato Etico Università degli Studi della Campania Luigi Vanvitelli” (approval No. 24997/2020) on 11 November 2020.

### 2.2. Patients Management and Follow-Up

Total body CT (computed tomography) scans for the initial staging of the disease were centralized at Centro Ames, accompanied by genetic assessment prior to commencing therapy, chosen through multidisciplinary consensus involving an oncologist, surgeon, and radiotherapist. The initial staging was conducted via CT imaging following the 8th edition of the TNM staging system for NSCLC by the Union for International Cancer Control (UICC) and the American Joint Committee on Cancer (AJCC). In surgically treated patients, staging was further validated through pathological examination, demonstrating a very high concordance between radiological and pathological findings. Subsequently, regardless of the therapeutic approach applied, patients underwent CT scans and regular clinical follow-ups every three months. However, in these cases, subsequent CT scans were permitted outside of Centro Ames. It is crucial to note that the primary objective of this study does not include assessing the response to therapy.

### 2.3. Genetic Assessment

Blood samples were collected before initiation of any therapy (from −14 to −1 days) using BCT^®^ cell-free DNA tubes (STRECK, La Vista, NE, USA). Plasma was obtained through standard centrifugation, aliquoted, and stored at −80 °C prior to analysis. Circulating free DNA (cfDNA) was extracted from plasma using the QIAamp DNA Blood Mini Kit (Qiagen, Hilden, Germany). Concentration, quantity, and integrity of cfDNA were assessed prior to utilization. The size distribution of cfDNA fragments was determined using a 2100 BioAnalyzer (Agilent Technologies, Santa Clara, CA, USA). For a comprehensive genetic assessment, we employed the TruSight™ Oncology 500 kit (Illumina, San Diego, CA, USA) to construct genomic libraries, targeting 523 cancer-relevant genes. This assay facilitates the detection of indels, small nucleotide variants (SNVs), splice variants, copy-number/structural variations, and gene fusions. Sequencing was conducted on the Illumina NovaSeq 6000 platform. The Illumina TruSight Oncology 500 bioinformatics pipeline was utilized for the analysis and in-house scripts for the interpretation of the sequencing results in this study. Each sample generated a median of 116 million reads, with coverage in the target region surpassing the manufacturer’s recommended threshold of 150×. Sequence data were aligned to the human reference genome GRCh37 using the Burrows–Wheeler Aligner with default parameters (http://www.ncbi.nlm.nih.gov/projects/genome/assembly/grc/human/index.shtml, last accessed on 4 February 2024). Variants were filtered using unmatched normal datasets and excluded if the global minor allele frequency was less than 1%. Gene variants were reported using a standardized nomenclature primarily based on protein-level amino acid sequence changes, with the prefix “p.”. In cases of non-coding or synonymous variations, the genomic change was documented.

### 2.4. Feature Extraction

We extracted radiomic features from each pretreatment CT image. The CT data, originally measured in Hounsfield units, were not converted into linear absorption coefficients because all scans were acquired using the same equipment. Prior to feature extraction, the CT volumes were resampled into cubic voxels with dimensions of 2 × 2 × 2 mm. The volume of interest (VOI) was manually delineated using ITK-SNAP (Insight Segmentation and Registration Toolkit-Segmentation and Navigation and Processing) v4.0, a software application for image segmentation and volume calculation (version 3.8.0, USA, http://www.itksnap.org/, last accessed in February 2024), by a single radiologist (RM) to mitigate intra-observer delineation variability. The VOI was set on the three-dimensional (3D) CT image for all patients, and then radiomic features depending on histogram (first-order) and texture (second-order: glcm, glrlm, glszm, ngtdm, and gldm) of the VOIs were extracted; each feature has been computed on original and wavelet (coif1) images. Thus, each case has 851 features extracted from original and wavelet-filtered images. Through the feature extraction process, the Pyradiomics (https://pyradiomics.readthedocs.io/, last accessed on 4 February 2024) package in the Python environment has been used. Furthermore, to avoid redundancy for such selected features, we used Pearson’s correlation coefficient analysis and limited the feature spaces by discarding features that were highly correlated with the others. The radiomic characteristics adhere to the specifications outlined in the Imaging Biomarker Standardization Initiative (IBSI) guidelines [17].

### 2.5. Feature/Gene Association Study

We assessed the correlation between individual features and gene variations utilizing the feature importance method. Following comprehensive deliberation, it was determined to exclusively analyze genetic variants exhibiting a frequency of genetic events greater than 3 out of 57 but less than 55. Variants deviating from these specified thresholds were considered non-contributory to identifying an association with radiomics and were consequently excluded from subsequent analyses. Specifically, for each radiomic feature, we computed the Area Under the Curve-Receiver Operating Characteristic (AUC-ROC) concerning each gene, treated as a categorical variable with two levels (mutated vs. non-mutated) using c-statistics. As commonly understood, the AUC-ROC ranges from 0.5 to 1, where 0.5 indicates a weak association and 1 signifies a robust association.

To illustrate the correlation between radiomic features and genetic factors, we employed a heatmap, i.e., each pixel represents a feature-gene pair, with the color denoting the strength of the association (red corresponds to 1, and white to 0.5). Successful associations were defined as AUC > 0.5 with *p* < 0.05, signifying statistical significance.

### 2.6. Machine Learning

In our exploratory investigation, we employed machine learning techniques to scrutinize the associations between radiomic features and genetics. The primary objective was to predict genetic mutations based on radiomic features. Specifically, we selected radiomic features characterized by minimal intercorrelation and fed them into a Support Vector Machine (SVM) classifier [18]. The genes identified in the feature/gene association study served as the target variables, acting as classification labels. To ensure the robustness of our findings, we implemented cross-validation, a technique that enhances the model’s reliability [19]. This method involves iteratively partitioning the dataset into subsets for both training and testing, validating the SVM classifier’s performance throughout the bootstrap process. The incorporation of cross-validation contributes to the overall rigor of our analysis, strengthening the validity of the identified associations between radiomic features and genetic mutations.

### 2.7. Sample Size, Statistical Analysis, and Data Presentation

This observational cohort study involves patients who underwent staging with CT before therapy and underwent genetic assessment through liquid biopsy. The primary objective is to elucidate any associations between radiological and genetic features using a radio-genomic combined approach. The study adheres to the principles outlined in the Declaration of Helsinki and is designed following the checklist recommended by the Strengthening the Reporting of Observational Studies in Epidemiology (STROBE) statement [20]. Approval for the study was obtained from the independent Internal Review Board (IRB) of Centro Ames, considering its non-scientific futility and aligning with methods to ensure patient privacy and data protection as per the General Data Protection Regulation. Due to the exploratory nature of this study, which aims not to develop a multivariate model but to explore associations between radiomic and genomic biomarkers, a power analysis for sample size was not conducted. The IRB imposed a maximum two-year duration and limited the sample size to 60 patients with a clear life expectancy exceeding three months, as assessed by three oncologists. These constraints consider logistical, resource, and practical aspects to ensure the feasibility and ethical implementation of the study within a specified timeframe and with a manageable cohort size.

The associations between specific gene variants and clinical and pathological variables were assessed using the χ^2^ test to determine their relationships. A significance level of *p* < 0.05 was considered statistically significant. For time-to-outcome analysis, overall survival (OS) was measured from the initiation of treatment until death from any cause. Progression-free survival was not included as an objective due to significant heterogeneity in treatments and follow-up radiologic reassessments. Instead, vital status was chosen as a more reliable and robust outcome for reporting and analysis. A descriptive prognostic analysis based on the identified genes was adopted to evaluate whether our radiomics model can predict a significant prognostic genetic feature. The Kaplan–Meier product limit method was utilized to create graphical representations of OS. Univariate analysis was conducted using the Log-rank test to assess differences in survival among various factors. The hazard ratio (HR) was used to estimate the probability of the endpoint (death) occurring at any given time for an individual with a specific risk factor compared to an individual without that risk factor. HRs were reported along with their corresponding 95% confidence intervals (CIs). Given the relatively small sample size and the exploratory nature of the study, no prior attempts were made to perform multivariate analysis. Statistical analysis was conducted using MedCalc^®^ 9.3.7.0 (www.medcalc.org, last accessed on 11 February 2024) and Excel software (Microsoft Excel 365).

## 3. Results

### 3.1. Clinicopathological Characteristics of the Analyzed Cohort

Following the study criteria, 66 patients underwent initial screening. Among them, six were excluded due to diagnostic uncertainty or the identification of a neuroendocrine phenotype, two for technical reasons related to the inability to obtain reliable genetic assessments, and one was lost to immediate follow-up. The clinical and pathological characteristics of the 57 analyzed patients diagnosed with NSCLC, comprehensively characterized from both radiomic and genomic perspectives, are detailed in Table 1.

Patients underwent characterization following cytological or histological diagnosis of NSCLC and prior to initiating any form of therapy. The age distribution exhibited a relatively balanced split, with 54.4% of patients below 70 years old and 45.6% aged 70 or above. Gender distribution revealed a predominance of males (68.4%). Histological examination unveiled a prevalent occurrence of adenocarcinoma (73.7%), while squamous cell carcinoma constituted the remaining 26.3%. Performance Status, evaluated by the ECOG scale, indicated a majority with a score of 0 (56.1%), followed by 31.6% with a score of 1, and a smaller fraction with a score of 2 (12.3%). The smoking history of participants displayed a diverse pattern, with only 8.8% classified as non-smokers (91.2% were former or current smokers). Disease staging demonstrated a considerable distribution across stages I/II (38.6%), III (31.6%), and IV (29.8%). Concerning the primary tumor, 57.9% presented with pT 1/2, 14.0% with pT 3, 19.3% with pT 4, and 8.8% were classified as non-definable. Regional lymph node involvement (pN) varied, with 56.1% having pN 0, 7.0% with pN 1, 24.6% with pN 2, 3.5% with pN 3, and 8.8% categorized as non-definable. Four patients presented with *EGFR*-mutated tumors. The front-line treatment modalities exhibited a physiologically heterogeneous landscape, encompassing both local definitive treatments and systemic therapies predominantly aligned with initial stages.

### 3.2. Molecular and Radiomic Profiling

**Figure 1:**
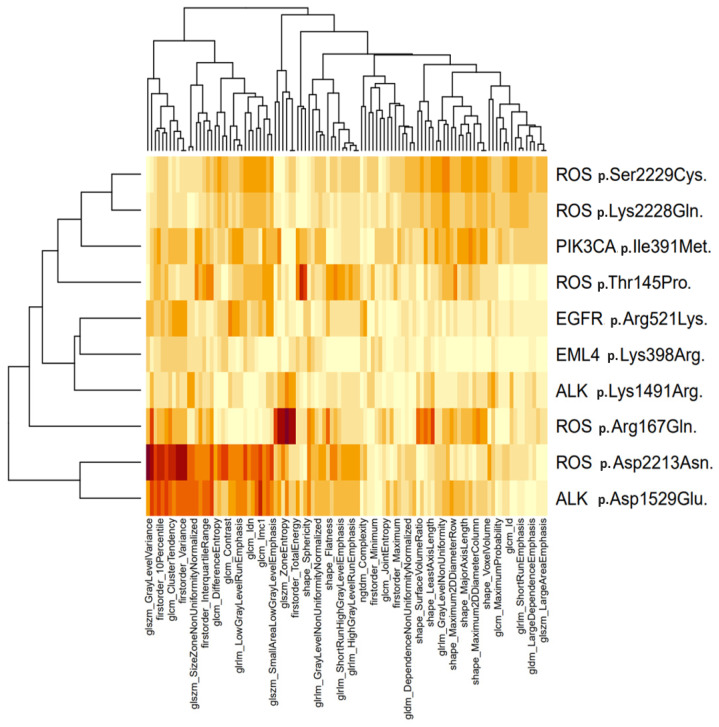
The figure displays a heatmap depicting the correlation between radiomic features and specific genetic variants. Each pixel represents a feature–gene pair, with color intensity ranging from white to increasingly intense red nuances, indicating the strength of the association. Significant associations, denoted by AUC > 0.5 with *p* < 0.05, are highlighted in red, representing statistical significance. Notably, distinct features show a substantial correlation with point mutations in *ROS1* p.Thr145Pro, *ROS1* p.Arg167Gln, *ROS1* p.Asp2213Asn, and *ALK* p.Asp1529Glu, while minimal correlation is observed for other genes.

Molecular and radiomic profiling were conducted to comprehensively characterize the tumors in the study cohort. Eight hundred fifty-one radiomic features were initially extracted for each of the selected patients. Concurrently, the relationship between radiomic features and genetic assessments was visualized in the heatmap presented in Figure 1.

Specific features are significantly associated with point mutations of *ROS1* p.Thr145Pro (shape_Sphericity, AUC-ROC: 0.68), *ROS1* p.Arg167Gln (glszm_ZoneEntropy, 0.76; firstorder_TotalEnergy, 0.89), *ROS1* p.Asp2213Asn (glszm_GrayLevelVariance, 0.79; firstorder_RootMeanSquared, 0.73), *ALK* p.Asp1529Glu (glcm_Imc1, 0.71), while for other genes, the correlation is negligible. No rearrangements of *ROS1* and *ALK* or pathogenic mutations of *BRAF* were identified in the analyzed series. The Support Vector Machine classifier algorithm is detailed in Supplementary File S1. Despite the molecular consequence of the chosen genetic variants being a “missense” alteration, only the *ALK* p.Asp1529Glu variant (ClinVar ID: 133476) is present in ClinVar. ClinVar offers a rigorous and comprehensive overview of the clinical significance of the associated genetic variant, providing valuable insights into its potential impact on health and disease. However, it is important to note that gene variants may not be included in the ClinVar database for various reasons, including insufficient available literature, unverified or conflicting information, and a lack of expert review.

### 3.3. Genetic Variants and Prognostic Analysis

**Figure 2:**
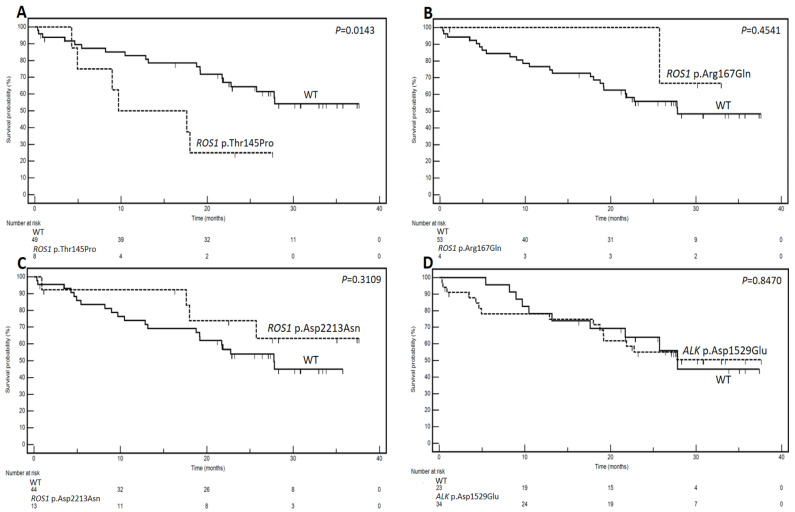
Kaplan–Meier survival curves delineate the impact of point mutations in *ROS1* p.Thr145Pro (**A**), *ROS1* p.Arg167Gln (**B**), *ROS1* p.Asp2213Asn (**C**), and *ALK* p.Asp1529Glu (**D**) on the survival of 57 analyzed patients with NSCLC. Log-rank test *p*-values are indicated on the respective curves. The number at risk for each group is reported below each panel.

With an exploratory aim, the prognostic role of the predicted gene variants was investigated. Figure 2 depicts the Kaplan–Meier curves, while Table 2 displays the results of the prognostic analysis related to the identified variants.

Notably, for *ROS1* p.Thr145Pro, the cohort with the mutation demonstrated a markedly shorter median survival of 9.7 months compared to the wild-type (WT) group, where the median survival was not reached (*p* = 0.0143). The hazard ratio (HR) was calculated at 5.35 (95% CI: 1.39–20.48). Conversely, no statistically significant associations with survival were observed for *ROS1* p.Arg167Gln, *ROS1* p.Asp2213Asn, and *ALK* p.Asp1529Glu (*p* = 0.4541, *p* = 0.3109, and *p* = 0.8470, respectively). These preliminary findings hint at a potentially substantial prognostic impact associated with *ROS1* p.Thr145Pro.

## 4. Discussion

In this study, we comprehensively characterized 57 patients diagnosed with NSCLC from both radiomic and genomic genetic perspectives. The analysis identified specific radiomic features associated with non-synonymous point mutations of *ROS1* (p.Thr145Pro, p.Arg167Gln, p.Asp2213Asn), and *ALK* (p.Asp1529Glu), suggesting potential molecular correlates. Interestingly, exploratory prognostic analysis revealed a significant association between the *ROS1* p.Thr145Pro mutation and shorter median survival compared to the wild-type group. This variant has been previously described in a study aimed at assessing the qualitative relationship between liquid biopsy and conventional tissue biopsy [21], but there are no background data regarding its prognostic significance.

NSCLC arises from a complex interplay of genetic, environmental, and lifestyle factors. The primary etiological factor is cigarette smoking, which contributes to the majority of NSCLC cases by inducing mutations in tumor suppressor genes and oncogenes involved in cell growth and apoptosis. Key driver mutations include *TP53*, *RB1*, *KRAS*, *STK11*, and *PIK3CA* [22]. Furthermore, mutations in *EGFR* and rearrangements of *ALK* and *ROS1* genes lead to constitutive activation of signaling pathways that promote cellular proliferation and survival. Rearrangements of *RET* and *NTRK* genes are less common. Aberrant epigenetic modifications, such as DNA methylation and histone acetylation, also influence the expression of genes involved in the pathogenesis of NSCLC. Knowledge of NSCLC genetics has revolutionized the treatment landscape for metastatic NSCLC over the past few decades. Patients with “oncogene-addicted” subtypes of NSCLC benefit substantially from tyrosine kinase inhibitors (TKIs), which specifically target these mutations and often result in improved response rates and time-to-outcome compared to traditional chemotherapy. Just to give a few examples, *EGFR* mutations can be effectively targeted with drugs like gefitinib, erlotinib, and osimertinib, while *ALK* rearrangements respond well to crizotinib, ceritinib, and alectinib. *ROS1* and *NTRK* rearrangements can be targeted by crizotinib and entrectinib, respectively. Inhibitors of *RET* fusion products are also available, such as selpercatinib [23,24]. In contrast, “non-oncogene-addicted” NSCLC, which lacks these driver mutations, has seen significant advancements with the introduction of immune checkpoint inhibitors. Agents such as pembrolizumab, nivolumab, and atezolizumab, which target the PD-1/PD-L1 pathway, have shown remarkable efficacy, particularly in patients with high PD-L1 expression. These immunotherapies can be used as monotherapy or in combination with chemotherapy, providing a new approach to improving survival outcomes [25]. Thus, genetic diagnostics are increasingly demanded and utilized for planning and predicting responses to treatments and prognosis [26].

In this context, the exploration of predicting cancer genetics through non-invasive radiomic techniques represents a cutting-edge frontier in scientific inquiry, currently undergoing thorough investigation [27]. Research into the utilization of conventional CT features and CT image-based radiomic features for forecasting the gene mutation status of lung cancer is still in its early stages. Additionally, these studies face inherent constraints, primarily stemming from substantial expenses, especially in cases involving extensive genetic profiling, as seen in our study, and the widespread unavailability of advanced technologies in global healthcare settings. Identifying specific features strongly associated with genetic alterations may, in some instances, facilitate narrowing the scope of assessments and reducing costs. The prediction of tumor genetics in radiomics relies on the presumption of conducting a non-invasive evaluation of molecular characteristics in tumor tissues, which can be challenging in certain tumor types, such as NSCLC. Therefore, in this context, we considered it pertinent to explore and generate hypotheses regarding the technical feasibility of identifying associations between genomics acquired through liquid biopsy assessments [28] and radiomics. One of the advantages of liquid biopsy, in addition to its non-invasive nature, is its high sensitivity, making it a candidate as a prognostic biomarker as well as a tool for detecting minimal residual disease (not clinically evident) and monitoring treatment response [29]. Potential issues of false negatives due to low tumor burden do not apply to our study, as the neoplastic disease was always radiologically visible in patients. Our scientific endeavor is in line with the current body of literature, although the majority of studies have not concentrated on individual genes; instead, they involve complex molecular signatures [30]. Additionally, some investigations have been limited to either early or advanced stages [31,32], while others do not correlate genetic and radiomic data with prognosis [33,34]. Despite the limitations of these data, radiomics holds promising potential in guiding treatment strategies and predicting survival benefits in NSCLC. By capturing intricate features from routine imaging scans, radiomics provides a unique, non-invasive, and comprehensive assessment of tumor heterogeneity. It facilitates the identification of subtle “imaging biomarkers” such as tumor shape, texture, and spatial relationships within the tumor and its surroundings. These biomarkers can stratify patients into different risk groups and predict individual responses to specific therapies. Moreover, radiomics-based models can integrate with molecular information (genetic characteristics), enhancing prognostic and predictive precision. Therefore, radiomics may contribute to personalized medicine by facilitating early detection of crucial biological features, monitoring disease heterogeneity, and optimizing therapeutic outcomes.

Our study highlights that certain genetic variants are associated with specific radiomic features, with one, *ROS1* p.Thr145Pro, potentially impacting prognosis. The *ROS1* gene encodes a receptor tyrosine kinase involved in cancer progression, particularly in NSCLC, where gene rearrangements can lead to oncogenic potential [35]. Despite ClinVar not reporting missense variations at position 145, which belongs to the first fibronectin type-III structural domain of the protein, potential consequences on kinase activity cannot be ruled out. Mutations within structural sites can significantly influence protein function, disrupting native conformation despite the unaltered catalytic region [36]. Unfortunately, classical *ROS1* rearrangements were not detected in our series, likely due to their low frequency and the non-selection of our cases for such alterations. Nevertheless, technically, it remains possible to identify associations between radiomic features and genetic alterations.

This exploratory and hypothesis-generating study motivates us to broaden the sample size and engage academic partners in a multi-centric context, wherein in vitro assessments will be incorporated to investigate the activity of altered genes.

## 5. Study Limitations

The limitations of this study warrant detailed discussion, with the foremost being the low sample size and the heterogeneity of treatments, although these are partially mitigated by the uniformity of technical assessments and the prospective nature of the study. It is evident that these limitations preclude drawing definitive conclusions. For transparency and scientific integrity, it is crucial to emphasize that patients with ROS1 p.Thr145Pro mutations amount to 8 out of 57. This number may be susceptible to variations and biases related to unknown factors. As detailed in Supplementary File S2, we examined potential associations between the presence of this genetic variant and known prognostic factors, revealing no significant imbalance. However, this does not protect against the possible detrimental effects of treatment heterogeneity, and more complex analyses, including multivariate analysis, would be difficult to conduct and justify. These reflections align with the exploratory nature of the study and necessitate caution in interpreting our results.

Furthermore, it is crucial to emphasize that, akin to the absence of *ROS1* and *ALK* rearrangements, no other actionable alterations, such as those in *EGFR*, were effectively linked with radiomics findings. For instance, only one variant of *EGFR* (ClinVar ID: 134021; *EGFR* p.Arg521Lys) surpassed the initial statistical criteria for a potential association (Figure 1), yet the predictive strength is too low to justify further analysis. This is certainly correlated with the documented low frequency of these alterations and the relatively limited sample size utilized in the study. While this does not compromise the scientific validity of our data, it distinctly diminishes the practical “utility” of the results in terms of predicting therapeutically targetable mutations. One approach to surmounting this limitation is to include in future case series only patients with actionable mutations in metastatic stages. This methodological choice, while obviously extending the study duration, naturally demands a multicentric effort.

Finally, although the case series is consecutive, an excess of early-stage cases (stages I/II) is observed. While this constitutes a positive phenomenon from a clinical–therapeutic perspective, it necessitates elucidation as it contrasts with an inverse phenomenon, wherein a higher incidence of advanced cases at onset (at least 70%, as reported in the literature) might be anticipated. The principal reason is attributed to the implementation of lung cancer screening programs among smoking individuals within the structures involved in this study. A portion of the patients is derived directly from these screening initiatives.

## 6. Conclusions

Radiomics represents a leading edge in NSCLC research, offering a non-invasive assessment of tumor heterogeneity. Integration with molecular data enhances prognostic precision, supporting personalized medicine by enabling early detection of biological features and optimizing prognostic and therapeutic outcomes. Our analysis identified specific radiomic features associated with non-synonymous point mutations in *ROS1* (p.Thr145Pro, p.Arg167Gln, p.Asp2213Asn) and *ALK* (p.Asp1529Glu), suggesting potential molecular correlations. Particularly notable was the finding from prognostic analysis, which revealed a significant association between the *ROS1* p.Thr145Pro mutation and shorter median survival compared to the wild-type group. Despite acknowledged limitations, our study provides valuable insights into the interplay between genomics and radiomics in NSCLC. The association between radiomic features and the *ROS1* p.Thr145Pro variant warrants further investigation due to its potentially significant prognostic implications.

## Figures and Tables

**Table 1:** Clinico-pathological characteristics of the analyzed series.

Characteristic	No.	%
Age		
<70	31	54.4
≥70	26	45.6
Gender		
Male	39	68.4
Female	18	31.6
Hystotype		
Adenocarcinoma	42	73.7
Squamous	15	26.3
PS ECOG		
0	32	56.1
1	18	31.6
2	7	12.3
Smoking		
Non smoker	5	8.8
Former smoker	27	47.4
Current smoker	25	43.8
Stage		
I/II	22	38.6
III	18	31.6
IV	17	29.8
T		
1/2	33	57.9
3	8	14.0
4	11	19.3
* Non-definable	5	8.8
N		
0	32	56.1
1	4	7.0
2	14	24.6
3	2	3.5
* Non-definable	5	8.8
*EGFR* mutation status		
Mutated	4	7.1
Wild-type	53	92.9
Type of front-line treatment		
Surgery	26	45.6
Platinum-based CT	10	17.5
Active palliative treatment	4	7.0
CT and/or RT followed by surgery	4	7.0
Target therapy	4	7.0
ICI monotherapy	3	5.3
Non-platinum based CT	3	5.3
Surgery followed by CT and/or RT	3	5.3

* Clinical and/or pathological T and N were not clearly definable in some cases because of tumor location, interobserver discordance, and ambiguities in clinical data leading to a final unresolved uncertainty.

**Table 2:** Univariate analysis of prognostic power of genetic variants associated with radiomic features in the analyzed series.

Gene Variant	Dicothomization	Median Survivals (Months)	No. of Events/Patients	HR	95% CI	*p* at Log-Rank Test
*ROS1* p.Thr145Pro	Mutated vs. WT	9.7 vs. NR	6/8 vs. 19/49	5.35	1.39–20.48	0.0143
*ROS1* p.Arg167Gln	Mutated vs. WT	NR vs. 27.8	1/4 vs. 24/53	0.57	0.13–2.44	0.4541
*ROS1* p.Asp2213Asn	Mutated vs. WT	NR vs. 27.7	4/13 vs. 21/44	0.62	0.25–1.55	0.3109
*ALK* p.Asp1529Glu	Mutated vs. WT	NR vs. 27.8	15/34 vs. 10/23	1.08	0.48–2.40	0.8470

CI: confidence interval; HR: hazard ratio; NR: not reached; WT: wild-type.

## Data Availability

The original contributions presented in the study are included in the article/Supplementary Materials, further inquiries can be directed to the corresponding author.

## Supplementary Materials

The following supporting information can be downloaded at: https://www.mdpi.com/article/10.3390/genes15060803/s1. Supplementary File S1: Support Vector Machine classifier algorithm; Supplementary File S2: Associations between *ROS1* p.T145P gene variant and clinico-pathological characteristics.
