# Supplementary material for: Associations between Radiomics and Genomics in Non-Small Cell Lung Cancer Utilizing Computed Tomography and Next-Generation Sequencing: An Exploratory Study
Source: Genes (Basel). 2024 Jun 18;15(6):803. doi: 10.3390/genes15060803 (PMC11202615; doi:10.3390/genes15060803)
Supplement: Supplementary file 1 [file genes-15-00803-s001.zip › genes-3039200-supplementary.pdf]

Supplementary File 1. Support Vector Machine classifier algorithm.

```
[1] "============================================"
[1] "** ROS.miss.42.43..Asp2213Asn. **"
Support Vector Machines with Linear Kernel

57 samples
27 predictors
 2 classes: 'NO', 'SI'

No pre-processing
Resampling: Cross-Validated (5 fold, repeated 20 times)
Summary of sample sizes: 45, 46, 45, 46, 46, 46, ...
Resampling results:

  Accuracy   Kappa
  0.6728939  0.09240316

Tuning parameter 'C' was held constant at a value of 1


[1] "============================================"
[1] "** ROS..miss.6.43..Arg167Gln. **"
Support Vector Machines with Linear Kernel

57 samples
27 predictors
 2 classes: 'NO', 'SI'

No pre-processing
Resampling: Cross-Validated (5 fold, repeated 20 times)
Summary of sample sizes: 46, 46, 45, 45, 46, 46, ...
Resampling results:

  Accuracy   Kappa
  0.6862529  0.2129848

Tuning parameter 'C' was held constant at a value of 1
```

```
[1] "=========================================="
[1] "** ALK.miss.29.29..Asp1529Glu. **"
Support Vector Machines with Linear Kernel

57 samples
27 predictors
 2 classes: 'NO', 'SI'

No pre-processing
Resampling: Cross-Validated (5 fold, repeated 20 times)
Summary of sample sizes: 46, 47, 45, 45, 45, 45, ...
Resampling results:

  Accuracy   Kappa
  0.5227121  0.01528081

Tuning parameter 'C' was held constant at a value of 1
```

```
[1] "=========================================="
[1] "** ROS.miss.5.43..Thr145Pro. **"
Support Vector Machines with Linear Kernel

57 samples
27 predictors
 2 classes: 'NO', 'SI'

Pre-processing: centered (27), scaled (27)
Resampling: Cross-Validated (5 fold, repeated 20 times)
Summary of sample sizes: 45, 46, 45, 47, 45, 47, ...
Resampling results:

  Accuracy   Kappa
  0.7395455  -0.004348662

Tuning parameter 'C' was held constant at a value of 1
```

Supplementary File 2. Associations between *ROS1* p.T145P gene variant and clinic-pathological characteristics.

	**Age (years)**		**Gender**		**Stages**		**Histotype**		**PS ECOG**	
**Gene variant**	<70	>70	M	F	Non-met	Met	Adeno	Squamous	0/1	2
Wild-type	28	21	34	15	36	13	36	13	43	6
ROS1 p.T145P	3	5	7	1	4	4	6	2	7	1
P at chi-square test		0.3053		0.2948		0.1824		0.9280		0.9839

Adeno: Adenocarcinoma; ECOG: Eastern Cooperative Oncology group; F: female; M: male; met: metastatic; PS: Performance Status.
